# The novel antipsychotic cariprazine stabilizes gamma oscillations in rat hippocampal slices

**DOI:** 10.1111/bph.14923

**Published:** 2020-02-19

**Authors:** Maria A. Meier, Clement E. Lemercier, Christoph Kulisch, Béla Kiss, Balázs Lendvai, Nika Adham, Zoltan Gerevich

**Affiliations:** ^1^ Institute of Neurophysiology Charité – Universitätsmedizin Berlin Berlin Germany; ^2^ Pharmacological and Drug Safety Research Gedeon Richter Plc Budapest Hungary; ^3^ External Science and Innovation Allergan Plc Madison New Jersey USA

## Abstract

**Background and Purpose:**

Gamma oscillations are fast rhythmic fluctuations of neuronal network activity ranging from 30 to 90 Hz that establish a precise temporal background for cognitive processes such as perception, sensory processing, learning, and memory. Alterations of gamma oscillations have been observed in schizophrenia and are suggested to play crucial roles in the generation of positive, negative, and cognitive symptoms of the disease.

**Experimental Approach:**

In this study, we investigated the effects of the novel antipsychotic cariprazine, a D_3_‐preferring dopamine D_3_/D_2_ receptor partial agonist, on cholinergically induced gamma oscillations in rat hippocampal slices from treatment‐naïve and MK‐801‐treated rats, a model of acute first‐episode schizophrenia.

**Key Results:**

The D_3_ receptor‐preferring agonist pramipexole effectively decreased the power of gamma oscillations, while the D_3_ receptor antagonist SB‐277011 had no effect. In treatment‐naïve animals, cariprazine did not modulate strong gamma oscillations but slightly improved the periodicity of non‐saturated gamma activity. Cariprazine showed a clear partial agonistic profile at D_3_ receptors at the network level by potentiating the inhibitory effects when the D_3_ receptor tone was low and antagonizing the effects when the tone was high. In hippocampal slices of MK‐801‐treated rats, cariprazine allowed stabilization of the aberrant increase in gamma oscillation power and potentiated resynchronization of the oscillations.

**Conclusion and Implications:**

Data from this study indicate that cariprazine stabilizes pathological hippocampal gamma oscillations, presumably by its partial agonistic profile. The results demonstrate *in vitro* gamma oscillations as predictive biomarkers to study the effects of antipsychotics preclinically at the network level.

**Abbreviations**
ACSF: artificial CSF
CI: confidence interval
Physo: physostigmine

**What is already known**

- Gamma oscillations are disturbed in patients with schizophrenia and in animal models of the disease.

**What does this study add**

- The novel antipsychotic cariprazine stabilized hippocampal gamma oscillations in rats pretreated with MK‐801.

**What is the clinical significance**

- *In vitro* gamma oscillations are predictive biomarkers to evaluate antipsychotics preclinically at the network level.

## 1 INTRODUCTION

To date, schizophrenia is understood as a neurodevelopmental disease arising from complex interactions between genetic and environmental factors, inducing changes in neuronal networks and alterations in multiple neurotransmitter systems (Birnbaum & Weinberger, 2017). The pathophysiology underlying the symptoms of schizophrenia remains poorly understood. Some of the molecular changes ultimately leading to the symptoms of the disease have been partially uncovered by the beneficial effects of antipsychotics. These drugs are used primarily to decrease the positive symptoms of the disease, which are mainly attributed to an excessive dopaminergic tone leading to overactivation of dopamine D_2_‐like receptors (Miyamoto, Miyake, Jarskog, Fleischhacker, & Lieberman, 2012). However, much less is known regarding the molecular and network backgrounds of negative and cognitive symptoms, as very few drugs with multiple receptor profiles have been associated with alleviation of these symptoms in the clinic.

https://www.guidetopharmacology.org/GRAC/LigandDisplayForward?ligandId=7671 (United States: Vraylar®; Europe: Reagila®) is a novel antipsychotic drug that was recently approved for the treatment of schizophrenia in adults (United States and Europe) and has shown treatment benefits against risperidone in patients with predominantly negative symptoms (Németh et al., 2017). Cariprazine is a high‐affinity https://www.guidetopharmacology.org/GRAC/ObjectDisplayForward?objectId=216‐preferring D_3_/https://www.guidetopharmacology.org/GRAC/ObjectDisplayForward?objectId=215 partial agonist with additional partial agonistic action at https://www.guidetopharmacology.org/GRAC/ObjectDisplayForward?objectId=1. It also exhibits high affinity for the https://www.guidetopharmacology.org/GRAC/ObjectDisplayForward?objectId=7 and moderate affinity for the https://www.guidetopharmacology.org/GRAC/ObjectDisplayForward?objectId=6 receptors, with an antagonistic profile at both of these receptors (Kiss et al., 2010). Cariprazine has been shown to occupy both D_2_ and D_3_ receptors in the brain of patients with schizophrenia, albeit with a preference for D_3_ receptors, with ED_50_ ratios in the threefold to sixfold range (Girgis et al., 2016). This is in contrast to currently available antipsychotic medications, such as https://www.guidetopharmacology.org/GRAC/LigandDisplayForward?ligandId=38 https://www.guidetopharmacology.org/GRAC/LigandDisplayForward?ligandId=96 and https://www.guidetopharmacology.org/GRAC/LigandDisplayForward?ligandId=47, which at antipsychotic‐effective doses show high occupancy of D_2_ receptors with no significant occupancy of D_3_ receptors (Graff‐Guerrero et al., 2009; Mizrahi et al., 2011). Therefore, studies have suggested that the superior efficacy of cariprazine against the negative symptoms of schizophrenia (Németh et al., 2017) may arise from its unique pharmacological profile, in particular, its high affinity for D_3_ receptors compared to other antipsychotics (Gyertyán et al., 2011; Kiss et al., 2010). However, the link between the unique pharmacological profile and clinical effectiveness is not yet fully understood because of a lack of pharmacological data at the system level. Investigation of the modulation of gamma oscillations has been considered a useful method to identify molecular mechanisms by which cariprazine influences the symptoms of schizophrenia (Ahnaou, Huysmans, Van de Casteele, & Drinkenburg, 2017; Schulz, Heidmann, et al., 2012).

Gamma oscillations are a measure of synchronous population activity at frequencies between 30 and 90 Hz (Fries, 2009) and are known to be generated by rhythmic firing of fast‐spiking parvalbumin‐containing perisomatic interneurons (Cardin et al., 2009). The power of gamma oscillations increases during sensory processing, perception, attention, working memory or long‐term memory representation in both animals (Lu, Jefferys, Toescu, & Vreugdenhil, 2011) and humans (Herrmann, Munk, & Engel, 2004; Rodriguez et al., 1999), indicating that gamma oscillations are a useful biomarker to investigate cognitive processes at the network level. Abnormal gamma oscillations have been described in several psychiatric diseases, including schizophrenia (Gonzalez‐Burgos, Cho, & Lewis, 2015; Uhlhaas & Singer, 2010). In particular, enhanced gamma oscillations have been observed in patients with hallucinations (Baldeweg, Spence, Hirsch, & Gruzelier, 1998) or with more severe positive symptoms (Spencer et al., 2004), whereas reduced gamma oscillations correlate with the negative and cognitive symptom scores of the disease (Cho, Konecky, & Carter, 2006; Lee, Kim, Kim, Kim, & Im, 2010).

NMDA receptor hypofunction has been implicated in the pathophysiology of schizophrenia (Jentsch & Roth, 1999). A single dose of a non‐competitive https://www.guidetopharmacology.org/GRAC/FamilyDisplayForward?familyId=75 antagonist, such as https://www.guidetopharmacology.org/GRAC/LigandDisplayForward?ligandId=4233 or phencyclidine, induces psychotic, negative and cognitive symptoms in healthy humans resembling those observed in schizophrenia (Newcomer et al., 1999). In addition, patients receiving NMDA antagonists have been reported to experience exacerbations of schizophrenia symptoms (Lahti, Weiler, Tamara Michaelidis, Parwani, & Tamminga, 2001). In rodents, NMDA receptor antagonists induce a schizophrenia‐like phenotype, including hyperlocomotion, stereotypies, disruptions in pre‐pulse inhibition, impairments in attention, social behaviour and cognitive deficits (Cadinu et al., 2017; Wiescholleck & Manahan‐Vaughan, 2013). In these animals, a single acute dose of an NMDA receptor antagonist induced a strong increase in gamma oscillations both in the hippocampus and neocortex (Kehrer et al., 2007; Lemercier, Holman, & Gerevich, 2017). Furthermore, a previous report suggested that a single acute dose of NMDA receptor antagonists mimics acute first‐episode schizophrenia, whereas chronic administration simulates the chronic symptoms of the disease with predominating negative symptoms (Jentsch & Roth, 1999).

Previous studies indicated the pivotal modulatory role of D_3_ receptors in hippocampal gamma oscillations *in vitro* (Lemercier, Schulz, Heidmann, Kovács, & Gerevich, 2016; Schulz, Heidmann, et al., 2012). With regard to the high preference of cariprazine for D_3_ receptors, we hypothesized that cariprazine modulates hippocampal gamma oscillatory activity and this effect may underlie its beneficial effects observed in schizophrenia. Therefore, to gain insight on its working mechanism at the network level, we investigated the effects of cariprazine on pharmacologically induced oscillatory activity in hippocampal slices from treatment‐naïve and https://www.guidetopharmacology.org/GRAC/LigandDisplayForward?ligandId=2403‐treated rats.

## 2 METHODS

### 2.1 Animals and slice preparation

Acute brain slices were prepared from 6‐ to 9‐week‐old (180–230 g) Wistar rats (*n* = 85) of either sex and own breed in accordance with the guidelines of the European Union (Directive 2010/63/EU), and the institutional guidelines were approved by the Berlin Animal Ethics Committee (Landesamt für Gesundheit und Soziales Berlin, G0437/12) as previously described (Schulz, Klaft, et al., 2012). The animals were kept under 12‐hr light/dark conditions and given food and water ad libitum. Animal studies are reported in compliance with the ARRIVE guidelines (Kilkenny, Browne, Cuthill, Emerson, & Altman, 2010) and with the recommendations made by the *British Journal of Pharmacology.* The animals were anaesthetized with https://www.guidetopharmacology.org/GRAC/LigandDisplayForward?ligandId=2505 (2.0–2.4 vol%) and then decapitated. MK‐801‐treated animals received intraperitoneally a single dose of 5 mg·kg^−1^ MK‐801 (diluted in 1 ml saline/100 g body weight) (Lemercier et al., 2017; Manahan‐Vaughan, von Haebler, Winter, Juckel, & Heinemann, 2008) 24 hr before slice preparation. Vehicle‐treated control animals received the same amount of saline (0.9% NaCl, 1 ml/100 g). MK‐801 induced hyperlocomotion and stereotypy, therefore experiments were not blind to the experimenter. The brains were removed and immediately submerged in an ice‐cold sucrose artificial CSF (ACSF) slicing solution containing 80‐mM NaCl, 2.5‐mM KCl, 3‐mM MgCl_2_, 0.5‐mM CaCl_2_, 25‐mM glucose, 85‐mM sucrose, 1.25‐mM NaH_2_PO_4_, and 25‐mM NaHCO_3_ (320–330 mOsm), enriched with carbogen (95% O_2_, 5% CO_2_). Further, 400‐μm‐thick horizontal slices containing the hippocampal formation were cut on a vibratome (DSK microslicer DTK‐1000, Dosaka, Japan). The slices were immediately transferred to an interface‐type recording chamber and perfused with a flow rate of 1.7 ml·min^−1^ with warm (36°C) and carbogenated ACSF containing 129‐mM NaCl, 3‐mM KCl, 21‐mM NaHCO_3_, 1.25‐mM NaH_2_PO_4_, 1.8‐mM MgSO_4_, 1.6‐mM CaCl_2_, and 10‐mM glucose. The slices were allowed to recover for at least 1 hr before starting the experiments.

### 2.2 Extracellular recordings

Local field potentials were recorded from the stratum pyramidale in the CA3b area of the hippocampus with glass pipettes filled with ACSF (resistance <3 MΩ) as described earlier (Çalışkan et al., 2015; Schulz, Klaft, et al., 2012). Recordings were amplified by a custom‐made amplifier, low‐pass filtered at 1 kHz and sampled at 5 kHz by a CED 1401 interface (Cambridge Electronic Design, Cambridge, UK).

### 2.3 Data analysis and statistics

The data and statistical analysis comply with the recommendations of the *British Journal of Pharmacology* on experimental design and analysis in pharmacology (Curtis et al., 2018). Experiments were designed to generate groups of equal size. Slices were randomly allocated to recording chambers and randomized into drug‐treated or control group before treatment. For blinding, operators and analysts were different persons. Group size selection was planned by the program G*Power3 (Faul, Erdfelder, Lang, & Buchner, 2007). Power spectra were calculated every 2 min with a 120‐s window throughout the recording. Based on these power spectra, peak power, peak frequency, half bandwidth, or quality factor (*Q* factor) of the oscillations were determined off‐line by using a custom‐made script for the Spike2 software (version 7.10, Cambridge Electronic Design, Cambridge, UK). The *Q* factor of the oscillation (Lemercier et al., 2017) was calculated by means of the following equation: *Q* = *f*_0_/*B*, where *f*_0_ is the peak frequency and *B* is the bandwidth at 50% of maximum peak power and was used instead of half bandwidth in experiments where oscillations desynchronized and altered their peak frequency. The *Q* factor describes the relative distribution of the frequencies around the peak frequency and is therefore independent of changes in peak frequency. Oscillation parameters show a high variability and were therefore normalized in every slice to a 10‐min baseline period before drug application, ACh/physostigmine (Physo) wash‐out, or corresponding time in control experiments. Statistical analysis was performed with GraphPad Prism (GraphPad Software Inc., San Diego, USA). The D'Agostino–Pearson normality test was used to test the Gaussian distribution of the data. Absolute power values displayed a log‐normal distribution and are therefore presented as geometric mean with its confidence interval (CI). All other data are presented as mean ± SEM. At least four animals were used in each experimental group. The group data subjected to statistical analysis have a minimum of *n* = 5 independent samples per group. Several slices of each brain were used for experiments but not more than one slice per hemisphere for each treatment group. No outliers were removed from the data. Statistical comparisons between drug‐induced effects and time‐matched control changes were performed using Student's unpaired *t‐* test. Absolute power values were compared using the non‐parametric Mann–Whitney *U* test. In all analyses, the significance level was set at *P* < .05.

### 2.4 Materials

https://www.guidetopharmacology.org/GRAC/LigandDisplayForward?ligandId=6598 (Physo), MK‐801, https://www.guidetopharmacology.org/GRAC/LigandDisplayForward?ligandId=953 and https://www.guidetopharmacology.org/GRAC/LigandDisplayForward?ligandId=143 were obtained from Tocris Bioscience (Bristol, UK). https://www.guidetopharmacology.org/GRAC/LigandDisplayForward?ligandId=294 was purchased from Sigma‐Aldrich (Taufkirchen, Germany) and cariprazine was provided by Gedeon Richter (Budapest, Hungary). Stock solutions were prepared in water, and the experimental compounds were further diluted in ACSF to reach the final concentrations. Cariprazine was applied at a concentration of 10 μM. Pharmacologically effective cariprazine plasma concentrations were 213 and 115 nM in rat and humans, respectively (Gyertyán et al., 2011; Mauri et al., 2018). Since brain concentrations were found to be min. 7.6‐fold higher than the corresponding plasma concentrations in rats (Gyertyán et al., 2011), we estimated a concentration range of 1–2 μM in brain during active behaviour. To reach this steady‐state concentration by diffusion into the slice held in the interface‐type chamber, a factor of 5 was used.

### 2.5 Nomenclature of targets and ligands

Key protein targets and ligands in this article are hyperlinked to corresponding entries in http://www.guidetopharmacology.org, the common portal for data from the IUPHAR/BPS Guide to PHARMACOLOGY (Harding et al., 2018), and are permanently archived in the Concise Guide to PHARMACOLOGY 2019/20 (Alexander, Christopoulos et al., 2019; Alexander, Kelly et al., 2019).

## 3 RESULTS

### 3.1 D_3_ receptor activation inhibits hippocampal gamma oscillations

**Figure 1:**
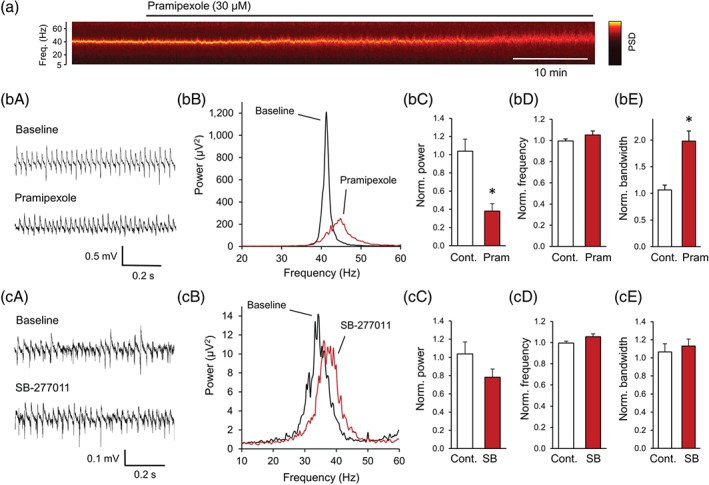
Activation of dopamine D_3_ receptors inhibits hippocampal gamma oscillations. (a) Spectrogram shows the inhibitory effect of pramipexole (30 μM), a D_3_ receptor‐preferring agonist, on gamma oscillations in the CA3 area induced by ACh (10 μM) and Physo (2 μM) in hippocampal slices. Brighter colours mean higher power values. (b) Original traces (left) and power spectra (right) of gamma oscillations before and after pramipexole application. (c) Bar diagrams show the effect of pramipexole (*n* = 7) on peak power, peak frequency and peak bandwidth compared to time‐matched parameters from control slices where only ACh and Physo were applied (*n* = 9). (d‐e) The D_3_ receptor antagonist SB‐277011 (30 μM) did not modulate hippocampal gamma oscillations. (d) Original traces (left) and power spectra (right) before and after SB‐277011 application. (e) Bar diagrams show the effect of SB‐277011 (*n* = 9) on peak power, peak frequency and peak bandwidth compared to time‐matched parameters from control slices (*n* = 9). Cont., control; Freq., frequency; Norm, normalized; Physo, physostigmine; Pram., pramipexole; PSD, power spectral density; SB, SB‐277011. Data are presented as mean ± SEM. ^*^ *P* < .05

Bath application of ACh (10 μM) and the acetylcholinesterase inhibitor physostigmine (2 μM) induced gamma oscillations in the CA3 area of the hippocampus which reached a plateau after ~90 min with a peak power of 463.6 μV^2^ (95% CI [183.5, 1171]), peak frequency of 39.1 ± 0.9 Hz and a half bandwidth of 5.6 ± 1.8 Hz (*n* = 19). The D_3_ receptor agonist pramipexole (30 μM; Mierau et al., 1995) significantly decreased the peak power of these oscillations to 38.1 ± 8.2% (P = .002; Figure 1a,b,c) and broadened the half bandwidth to 198.1 ± 19.1% (P = .002), whereas it did not significantly affect the peak frequency of the gamma oscillations.

The dopamine D_3_ receptor antagonist SB‐277011 (30 μM) did not affect the power, peak frequency or half bandwidth compared to control gamma oscillations in the hippocampus (Figure 1d,e).

### 3.2 Cariprazine increases the periodicity of non‐saturated hippocampal gamma oscillations

**Figure 2:**
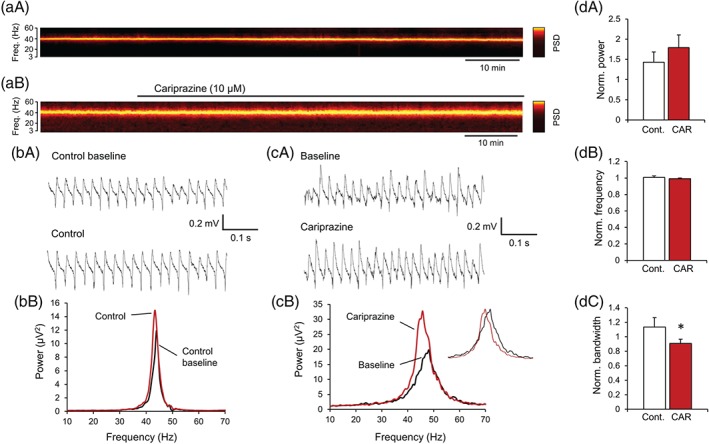
Cariprazine increases the periodicity of non‐saturated hippocampal gamma oscillations. (a‐b) Spectrograms show gamma oscillations in the CA3 area induced by non‐saturating concentrations of ACh (5 μM) and Physo (1 μM) without (a) and with cariprazine (10 μM) application (b). Brighter colours mean higher power values. (c) Original traces (top) and power spectra (below) of induced gamma oscillations in control slices after gamma oscillation induction (baseline) and 60 min later (control). (d) Original traces (top) and power spectra (below) of induced gamma oscillations before (baseline) and after cariprazine (10 μM) application. The inset shows the same power spectra rescaled to demonstrate the effect of cariprazine on the half bandwidth. (e‐g) Bar diagrams show the effect of cariprazine (*n* = 7) on peak power (e), peak frequency (f) and peak bandwidth (g) compared to time‐matched parameters from control slices (*n* = 7). CAR, cariprazine; Cont., control; Freq., frequency; Norm, normalized; Physo, physostigmine; PSD, power spectral density. Data are presented as mean ± SEM. ^*^ *P* < .05

Next, we investigated the effect of cariprazine on hippocampal gamma oscillations. Cariprazine (10 μM), similar to the D_3_ receptor antagonist SB‐277011, did not change any oscillation parameters when the oscillations were induced by saturating concentrations of ACh (10 μM) and physostigmine (2 μM; peak power: 134.9 ± 14.7%, *n* = 12, vs. 157.8 ± 33.1% with control, *n* = 7, *P* = .477; peak frequency: 98.2 ± 1.9% vs. 99.2 ± 1.1% with control, *P* = .720; half bandwidth: 109.1 ± 8.7% vs. 96.3 ± 6.6% with control, *P* = .321; data not shown). To test whether non‐saturated gamma oscillations were differentially affected by cariprazine, we induced oscillations with lower concentrations of ACh (5 μM) and physostigmine (1 μM, Figure 2). These oscillations developed with a significantly lower peak power (43.7 μV^2^, 95% CI: 19.2 to 99.1, n = 19, P = .015) and a higher peak frequency (45.9 ± 1.1 Hz, P < .001), while the half bandwidth remained unchanged (5.9 ± 0.6 Hz), consistent with previous observations with https://www.guidetopharmacology.org/GRAC/LigandDisplayForward?ligandId=298‐induced oscillations (Fisahn, Pike, Buhl, & Paulsen, 1998). Similar to the saturated gamma oscillations, cariprazine (10 μM) did not significantly modulate the power and peak frequency of these oscillations (Figure 2d,e,f) compared to the changes with control during the same time interval. However the half bandwidth of the non‐saturated oscillations was significantly reduced to 90.7 ± 5.9% (P = .044 compared to the control change: 113.4 ± 8.2%, Figure 2d,g).

### 3.3 Cariprazine shows a partial agonistic profile when modulating gamma oscillations

**Figure 3:**
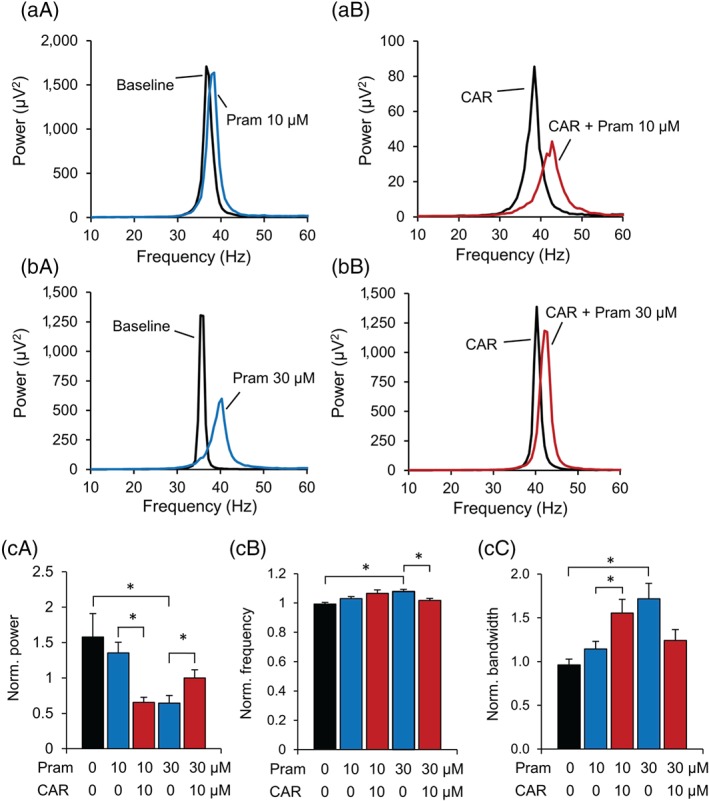
Cariprazine demonstrates a partial agonistic profile when modulating gamma oscillations. (a) Cariprazine (10 μM) potentiated the weak inhibitory effect of low‐concentration pramipexole (10 μM) on gamma oscillations. Power spectra show the effect of low‐concentration pramipexole in the absence (left) and presence (right) of cariprazine (10 μM). (b) Cariprazine antagonized the inhibitory effect of high‐concentration pramipexole (30 μM) on gamma oscillations. Power spectra show the effect of high‐concentration pramipexole in the absence (left) and presence (right) of cariprazine (10 μM). (c‐e) Bar diagrams show the modulation of pramipexole effects (*n* = 6–9) by cariprazine (*n* = 6) on peak power (c), peak frequency (d) and peak bandwidth (e). CAR, cariprazine; Norm, normalized; Pram., pramipexole. Data are presented as mean ± SEM. ^*^ *P* < .05

To evaluate how cariprazine interacts with the D_3_ receptor to modulate gamma oscillations depending on the current level of D_3_ receptor activation, we applied cariprazine prior to pramipexole treatment that was administered at two different concentrations. The low concentration of pramipexole (10 μM) did not significantly decrease the oscillation power, although a slight and non‐significant reduction was observed (Figure 3a,c). Cariprazine, however, potentiated the effect of 10‐μM pramipexole (Figure 3a,c).

On the contrary, the higher concentration of pramipexole (30 μM) decreased the power (*P* = .011) and increased the half bandwidth (*P* = .003), as well as the peak frequency (*P* < .001; Figure 3b‐e). Cariprazine (10 μM) inhibited the effect of pramipexole on the peak power (P = .048) and peak frequency (P = .016) whereas the effect on the half bandwidth was not significant (Figure 3b‐e). Thus, while cariprazine augmented the effect of the agonist at low D_3_ receptor stimulation, it had an antagonistic effect when the agonist tone was high.

### 3.4 Cariprazine does not affect the temporal dynamics of hippocampal gamma oscillations in non‐treated animals

**Figure 4:**
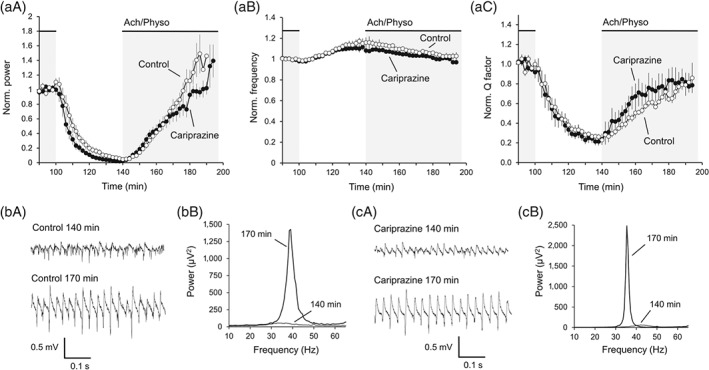
Cariprazine does not affect the temporal dynamics of hippocampal gamma oscillations in non‐treated animals. (a‐c) Gamma oscillations decayed after wash‐out of ACh (10 μM) and Physo (2 μM) and resynchronized again after repeated wash‐in in control experiments. Cariprazine (*n* = 6) did not affect the decay and resynchronization of peak power (a), peak frequency (b), and *Q* factor (c) compared to control (*n* = 6). Grey area indicates the presence of ACh/Physo in the slice. (d) Original traces (left) and power spectra (right) of gamma oscillations 40 min after wash‐out of ACh/Physo (140 min after the first application) and 30 min after the repeated wash‐in (170 min after the first application). (e) Original traces (left) and power spectra (right) of gamma oscillations in the presence of cariprazine 40 min after wash‐out of ACh/Physo (140 min after the first application) and 30 min after the repeated wash‐in (170 min after the first application). Norm, normalized; Physo, physostigmine; *Q* factor, quality factor

Neural network activity is dynamic and characterized by the appearance and disappearance of synchronization in time. Therefore, we investigated whether cariprazine was capable of modulating gamma activity in the temporal domain. We induced gamma oscillations in the hippocampus, followed by interruption of the ACh/physostigmine application for 40 min after 100 min of perfusion. Following wash‐out of ACh/physostigmine, the gamma oscillations desynchronized, as observed by a significant decrease in the power to 6.6 ± 2.1% (Figure 4a,d), while the peak frequency increased by 15.9 ± 4.4% (Figure 4b). Because of this shift in frequency, we expressed the spectral bandwidth as the *Q* factor expressing the relative distribution of the frequencies around the particular peak frequency, as described in Section 2. The *Q* factor significantly decreased during the wash‐out of ACh/physostigmine to 27.1 ± 2.4% (Figure 4c). After repeated wash‐in, gamma oscillations resynchronized: The power increased again to 69.2 ± 6.9% of the baseline, 30 min after repeated wash‐in, and the *Q* factor increased to 61.5 ± 3.9% during the same time interval. The peak frequency of the oscillations also decreased back to 107.5 ± 1.3%. Cariprazine (10 μM) did not significantly influence the temporal dynamics of the oscillations (Figure 4a,e). With cariprazine treatment, the power reached during the 30 min resynchronization period 62.0 ± 10.1% of the baseline (Figure 4a,e), while the *Q* factor increased to 74.3 ± 11.8% (Figure 4c) and the peak frequency decreased to 103.8 ± 1.4% (Figure 4b).

### 3.5 Cariprazine stabilizes hippocampal gamma oscillations in MK‐801‐treated rats

**Figure 5:**
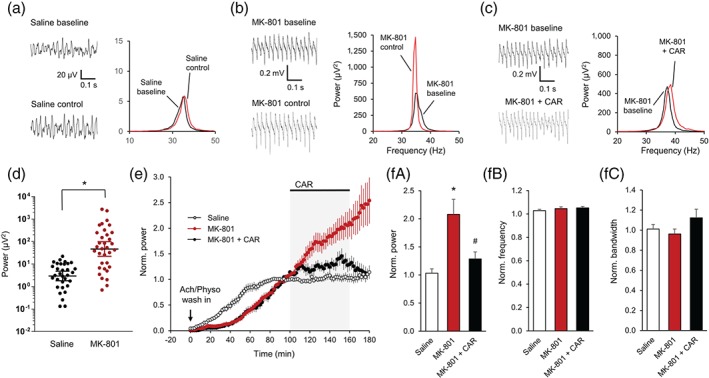
Cariprazine stabilizes gamma oscillations in MK‐801‐treated animals. (a‐b) Original traces (left) and power spectra (right) of ACh/Physo‐induced gamma oscillations in slices from saline‐ (a) or MK‐801‐treated rats (b) after gamma oscillation induction (baseline) and 60 min later (control). (c) Original traces (left) and power spectra (right) of induced gamma oscillations in slices from MK‐801‐treated rats before (baseline) and after cariprazine (10 μM) application. (d) Power of gamma oscillations 90 min after induction by ACh/Physo in saline‐ (*n* = 32) and MK‐801‐treated animals (*n* = 34). (e) Normalized power of gamma oscillations after application of ACh/Physo in saline‐ (*n* = 32) and MK‐801‐treated animals (*n* = 34) and the effect of cariprazine (*n* = 12) in MK‐801‐treated rats. (*f*) Bar diagrams show the effect of MK‐801 and cariprazine on the normalized peak power, peak frequency, and half bandwidth. CAR, cariprazine; Norm, normalized; Physo, physostigmine. Data are presented as mean ± SEM. ^*^ *P* < .05 compared to saline treatment, ^#^ *P* < .05 compared to MK‐801 treatment alone

In the next series of experiments, we investigated whether cariprazine affects disturbed gamma oscillations. To accomplish this, the effects of cariprazine were studied in hippocampal slices of rats that received a single dose of MK‐801, a model of first‐episode schizophrenia (Manahan‐Vaughan et al., 2008; Wiescholleck & Manahan‐Vaughan, 2013). In line with previous results (Lemercier et al., 2017), the hippocampal gamma oscillations induced by bath application of ACh (10 μM) and physostigmine (2 μM) had a significantly higher peak power after 90 min of induction compared to saline‐treated animals (P < .001, Figure 5a,b,d) and a broader half bandwidth (MK‐801: 5.19 ± 0.64 Hz, *P* = .033, compared to saline‐treated animals: 3.55 ± 0.37 Hz; not shown); however, the *Q* factor of the oscillations did not change (MK‐801: 9.9 ± 1.0, *P* = .103, compared to saline treatment: 12.5 ± 1.2; not shown). Similarly, the peak frequency of the oscillations was not significantly altered (MK‐801: 35.1 ± 1.1 Hz, *P* = .196, compared to saline‐treated animals: 33.3 ± 0.7 Hz, not shown). MK‐801 treatment affected the temporal dynamics of the oscillatory activity. The power of the oscillations did not stabilize after approximately 90 min but continuously increased to 208.1 ± 26.7% after 160 min of induction which is a clear difference compared to saline‐treated control animals where the power remained stable (103.3 ± 7.8%, P < .001, Figure 5e,f). Cariprazine (10 μM) applied for 60 min after 100‐min induction in MK‐801‐treated animals prevented the continuous increase in the power compared to the MK‐801 control change (128.6 ± 12.6%, P = .010, Figure 5e,f). In contrast, the bandwidth and frequency of the oscillations remained constant during this time interval and cariprazine did not significantly change any of the parameters (Figure 5f).

### 3.6 Cariprazine accelerates the temporal dynamics of gamma oscillations in the hippocampus of MK‐801‐treated animals

**Figure 6:**
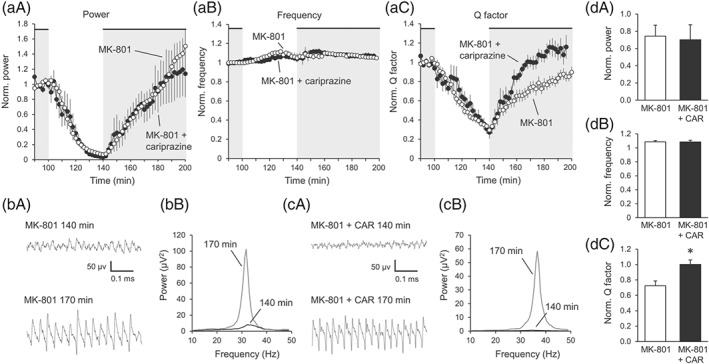
Cariprazine accelerates the temporal dynamics of gamma oscillations in the hippocampus of MK‐801‐treated animals. (a‐c)Cariprazine (*n* = 8) did not affect the dynamics of peak power (a) and peak frequency (b) but speeded up the resynchronization of the *Q* factor (c) compared to control (*n* = 21). Grey area indicates the presence of ACh/Physo in the slice. (d) Original traces (left) and power spectra (right) of gamma oscillations 40 min after wash‐out of ACh/Physo (140 min after the first application) and 30 min after the repeated wash‐in (170 min after the first application). (e) Original traces (left) and power spectra (right) of gamma oscillations in the presence of cariprazine 40 min after wash‐out of ACh/Physo (140 min after the first application) and 30 min after the repeated wash‐in (170 min after the first application). (f‐h) Bar diagrams show the effect of cariprazine on the peak power (f), peak frequency (g) and peak bandwidth (h) of resynchronized gamma oscillations 30 min after re‐application of ACh/Physo (170 min) compared to time‐matched parameters from control slices. CAR, cariprazine; Physo; physostigmine, Norm, normalized; *Q* factor, quality factor. Data are presented as mean ± SEM. ^*^ *P* < .05

Wash‐out of ACh/physostigmine gradually desynchronized gamma oscillations in MK‐801‐treated rats (Figure a‐d). The power of the oscillations decreased to 8.5 ± 1.1% after 40 min. During the same time, peak frequency showed a non‐significant trend to increase and the *Q* factor significantly decreased to 34.5 ± 2.7% (Figure 6c). Repeated wash‐in of ACh and physostigmine resynchronized gamma oscillations by increasing the power to 65.8 ± 11.9% and the *Q* factor to 66.5 ± 6.1% (Figure 6a,c). The peak frequency further increased to 109.1 ± 1.9% of the baseline (Figure 6b,g). Cariprazine did not influence the dynamics of the peak power (Figure 6a,f). The oscillations achieved in the presence of cariprazine 30 min after re‐application of ACh/physostigmine 69.9 ± 15.8% of their initial power. Similarly, the peak frequency further increased and was comparable to the changes with control (Figure 6b,g). However, cariprazine clearly and significantly accelerated the increase in *Q* factor after repeated administration of ACh/physostigmine reaching 97.9 ± 4.8% of the original baseline (P = .006, Figure 6c,h), thereby increasing the quality of oscillations during the resynchronization period.

## 4 DISCUSSION

The main findings of the present study are that the novel antipsychotic cariprazine, (a) does not modulate strong gamma oscillations but slightly improves the periodicity of moderate and unsaturated gamma activity, (b) behaves as a partial agonist at the network level when challenged with the D_3_ receptor agonist pramipexole, (c) stabilizes the power of gamma oscillations in MK‐801‐treated rats and (d) accelerates the resynchronization of gamma oscillations in MK‐801‐treated animals.

The present results derived from application of pramipexole alone demonstrate that stimulation of D_3_ receptors consistently decreases gamma activity in the hippocampus. These data are in line with our previous observations from other agonists showing that activation of D_3_ receptors decreases the power of gamma oscillations and selective D_3_ receptor antagonists prevent this effect (Lemercier et al., 2016; Schulz, Heidmann, et al., 2012). In slices from healthy brains, cariprazine only affected weak gamma oscillations induced by lower and non‐saturating concentrations of ACh by reducing the half bandwidth of these oscillations. There were no significant effects on strong and saturated gamma oscillations. These results suggest that cariprazine increases the synchronization of not fully developed oscillations by improving their periodicity towards the dominating frequency. The increased synchronization might support communication between cells and cell assemblies.

To investigate the D_3_ receptor partial agonist property of cariprazine, we modelled pharmacologically different D_3_ receptor tones by applying pramipexole at moderate (10 μM [low D_3_ receptor tone]) or high (30 μM [high D_3_ receptor tone]) concentrations and applied cariprazine under these conditions. Such an experimental paradigm might be considered a model of endogenous “selective” D_3_ receptor activation. In these pramipexole challenge experiments, we confirmed that cariprazine behaves as a D_3_ receptor partial agonist, namely under low D_3_ receptor tone cariprazine acts predominantly as a D_3_ receptor agonist, enhancing D_3_ receptor activation and decreasing gamma oscillations. On the contrary, under high D_3_ receptor tone cariprazine behaves as a D_3_ receptor antagonist and attenuates the inhibitory effects of high D_3_ receptor activation on gamma oscillations. Direct enhancement or reduction of gamma oscillations under control conditions could be counterproductive because they may lead to psychiatric symptoms, as shown in studies in patients with various psychiatric diseases and corresponding gamma oscillation alterations (Dickinson, Bruyns‐Haylett, Jones, & Milne, 2015; Herrmann & Demiralp, 2005; Uhlhaas & Singer, 2015). Our understanding of the function and role of dopamine D_3_ receptors in schizophrenia pathophysiology is rather limited. D_3_ receptors have a high affinity for dopamine (Sokoloff, Giros, Martres, Bouthenet, & Schwartz, 1990) and are suggested to be activated by volume transmission during the slower tonic dopamine release from dopaminergic fibres (Grace, 2016), and control the amplitude of the fast, phasic dopamine release during behaviourally salient stimuli (Sokoloff and Le Foll, 2017). In schizophrenia, this fine control of the phasic dopamine release amplitude is disturbed, resulting in a state of aberrant salience (Kapur, 2003). Our findings suggest beneficial effects of the partial agonistic characteristic of cariprazine via normalization of the disturbed gamma oscillations by stabilizing the dopamine effects in the hippocampus.

The normalizing effect of cariprazine at the level of neural networks, which was apparent at stable oscillations, could also be observed in pathological hippocampal networks. MK‐801 is considered as one of the most reliable models to induce a disease‐like phenotype in animals, including the negative and cognitive symptoms of schizophrenia (Cadinu et al., 2017; Meltzer et al., 2013). Hippocampal gamma oscillations in these animal models were found to be abnormally increased both *in vivo* (Kittelberger, Hur, Sazegar, Keshavan, & Kocsis, 2012) and *in vitro* (Kehrer et al., 2007; Lemercier et al., 2017). These electrophysiological abnormalities of gamma oscillations were suggested to underlie the symptoms of the disease (Gonzalez‐Burgos et al., 2015; Uhlhaas & Singer, 2010) and have been shown to be modulated by at least some of the available antipsychotics (Hudson, Rind, O'Brien, & Jones, 2016; Schulz, Heidmann, et al., 2012). In our *in vitro* experiments, following MK‐801 pretreatment, the oscillations did not reach a plateau and continuously increased until the end of the measurement. Application of cariprazine, acting as a partial agonist at the D_3_ receptors, effectively stabilized the unbridled oscillations and prevented the network from the deleterious effects of MK‐801 pretreatment (Figure 5c). This balancing activity could be one important mechanism by which cariprazine reaches its beneficial behavioural effects during positive and negative symptoms.

The temporal dynamics of gamma oscillatory activity in slices can be considered a plastic capability of the network. The second induction of gamma oscillations in the same network occurs significantly faster indicating that plastic changes had happened during the first induction and the circuitry remembers earlier oscillations (Zarnadze et al., 2016). This paradigm might be a physiological model of synaptic plasticity at the network level rather than selective electrical stimulation of one of the inputs at a chosen frequency. The speed of the resynchronization of gamma oscillations thus indicates the extent of activity‐dependent network modification in the hippocampus within a given time. Acceleration of this resynchronization may therefore indicate a dynamic network reorganization, which may be of benefit to cognitive functions (Reinhart & Woodman, 2014). In earlier observations, antagonism at D_3_ receptors was found to speed up the second synchronization (Lemercier et al., 2016), suggesting that D_3_ receptors have negative effects on neuronal plasticity at the network level and antagonism at these receptors may support plasticity within the network. These data may provide one possible explanation as to why D_3_ receptor knockout animals perform better in cognitive tests and why D_3_ receptor antagonists have pro‐cognitive effects in animal models (Nakajima et al., 2013). The partial agonist cariprazine did not alter the dynamics in control animals but significantly accelerated the resynchronization in MK‐801‐treated animals as measured by means of the *Q* factor. The *Q* factor (as described in Section 2) describes the distribution of the frequencies around the peak and herewith the periodicity of the oscillations (Lemercier et al., 2017). After wash‐out of ACh, the gamma oscillations desynchronized, as evident from the decreasing power and *Q* factor values. The resynchronization occurred significantly faster in the presence of cariprazine but only in MK‐801‐treated rats. Thus, these results suggest that cariprazine may have no effect on cognitive functions in healthy animals but could improve cognitive symptoms in schizophrenia models. In line with this, cariprazine has been shown to be effective against these domains in animal models (Barnes et al., 2018; Neill et al., 2016; Watson et al., 2016; Zimnisky et al., 2013) and in patients with schizophrenia (Daniel et al., 2017).

D_3_ receptors are expressed in the rat and human cortex (Bouthenet et al., 1991; Khan et al., 1998; Richtand, Kelsoe, Segal, & Kuczenski, 1995). In the hippocampus, the expression of the D_3_ receptor mRNA was found in the pyramidal cell layer of the cornu ammonis (Bouthenet et al., 1991) and the receptor protein on dendrites in both the stratum oriens and radiatum (Khan et al., 1998), suggesting the presence of D_3_ receptors on pyramidal cell dendrites. Although the expression of the dopamine D_3_ receptor is low in these regions, it seems comparable to that of D_2_ receptors (Khan et al., 1998). Additionally, the D_3_ receptor has the highest affinity for dopamine amongst all dopamine receptor subtypes, particularly when it is uncoupled from G proteins (Sokoloff et al., 1990; Van Tol et al., 1991). There are several possible cellular mechanisms by which D_3_ receptors on pyramidal cells can modulate local neuronal oscillations. In prefrontal pyramidal cells and auditory brainstem interneurons, D_3_ receptor activation was shown to decrease the excitability by inhibition of calcium influx via low‐voltage‐activated Cav3.2 calcium channels localized to the axon initial segment (Bender, Ford, & Trussell, 2010; Clarkson, Liptak, Gee, Sohal, & Bender, 2017). D_3_ receptors were also found to reduce inhibitory synaptic inputs in CA1 pyramidal cells by inducing endocytosis of GABA_A_ receptors (Swant, Stramiello, & Wagner, 2008). Since synchronous perisomatic inhibition by fast‐spiking interneurons is thought to be responsible for the generation of the gamma oscillations, reduction of the synchronizing currents by D_3_ receptors could explain the gamma‐inhibiting effects of these receptors.

Although our experiments with the D_3_ receptor agonist pramipexole and cariprazine indicated that cariprazine modulates gamma oscillations by acting on D_3_ receptors, antipsychotics often display a wide pharmacological profile with effective interactions on multiple receptors. In case of cariprazine, it has been reported that it possesses also partial agonistic and antagonistic actions at 5‐HT_1A_, 5‐HT_2A_ and 5‐HT_2B_ receptors, respectively (Kiss et al., 2010). Among them, the 5‐HT_1A_ receptors (Krause & Jia, 2005) were found to modulate gamma oscillations in the hippocampus suggesting that besides the D_3_ receptors, these receptors might also be involved in the mechanisms of action of cariprazine. Further investigations are needed to fully characterize the contribution of other receptors targeted by cariprazine in the modulation of gamma oscillations.

In summary, our results showed a dopamine D_3_ receptor partial agonistic profile for cariprazine at the network level by studying oscillatory activities. Additionally, cariprazine had a clear stabilizing effect on pathological hippocampal gamma oscillations in an NMDA hypofunction model of schizophrenia, which can be explained by its partial agonistic profile. Considering that perturbations in gamma oscillations have been suggested to be relevant to the positive, negative, and cognitive symptoms of schizophrenia, the ability of cariprazine to normalize gamma oscillations in the acute MK‐801 model of schizophrenia might further explain the clinical effectiveness of this novel agent. Additional experiments in the future are warranted to investigate the effects of cariprazine in the chronic MK‐801 model. The presented findings demonstrate the predictive validity of *in vitro* gamma oscillations as biomarkers to study the effects of antipsychotics preclinically at the network level.

## AUTHOR CONTRIBUTIONS

M.A.M., C.E.L. and C.K. performed the experiments and analysed the data. B.K., B.L. and N.A. actively contributed to designing the experiments and interpretation of data. Z.G. designed the experiments and analysed and interpreted the data. All authors drafted the manuscript.

## CONFLICT OF INTEREST

The study was supported by a grant from Allergan Plc and Gedeon Richter Plc. Allergan Plc and Gedeon Richter PLc were involved in the study design, analysis, interpretation of data, the decision to present results, and writing of manuscript. Charité – Universitätsmedizin Berlin was involved in the study design, all experimental parts, report and discussion of the results, and writing the manuscript. B.L. and B.K. are full‐time employees at Gedeon Richter Plc. N.A. is a full‐time employee at Allergan. M.A.M., C.E.L., C.K. and Z.G. are affiliated at Charité – Unversitatsmedizin Berlin.

## DECLARATION OF TRANSPARENCY AND SCIENTIFIC RIGOUR

This Declaration acknowledges that this paper adheres to the principles for transparent reporting and scientific rigour of preclinical research as stated in the *BJP* guidelines for https://bpspubs.onlinelibrary.wiley.com/doi/abs/10.1111/bph.14207, and https://bpspubs.onlinelibrary.wiley.com/doi/abs/10.1111/bph.14207 and as recommended by funding agencies, publishers and other organisations engaged with supporting research.

## Data Availability

Data reported in this manuscript are available within the article. Additional data may be requested at http://www.allerganclinicaltrials.com/PatientDataRequest.htm.

## References

[bph14923-bib-0001] Ahnaou, A. , Huysmans, H. , Van de Casteele, T. , & Drinkenburg, W. H. I. M. (2017). Cortical high gamma network oscillations and connectivity: A translational index for antipsychotics to normalize aberrant neurophysiological activity. Translational Psychiatry, 7(12), 1285 10.1038/s41398-017-0002-9 29249806 PMC5802558

[bph14923-bib-0002] Alexander, S. P. H. , Christopoulos, A. , Davenport, A. P. , Kelly, E. , Mathie, A. , Peter, J. A. , … CGTP Collaborators (2019). The concise guide to pharmacology 2019/2020: G protein‐coupled receptors. British Journal of Pharmacology, 176, S21–S141. 10.1111/bph.14748 31710717 PMC6844580

[bph14923-bib-0003] Alexander, S. P. H. , Keely, E. A. , Mathie, A. , Peter, J. A. , Veale, E. L. , Armstrong, J. H. , … GTP collaborators (2019). The concise guide to pharmacology 2019/2020: Introduction and other protein target. British Journal of Pharmacology, 176, S1–S20. 10.1111/bph.14747 31710719 PMC6844537

[bph14923-bib-0004] Baldeweg, T. , Spence, S. , Hirsch, S. R. , & Gruzelier, J. (1998). Gamma‐band electroencephalographic oscillations in a patient with somatic hallucinations. Lancet, 352(9128), 620–621. 10.1016/S0140-6736(05)79575-1 9746027

[bph14923-bib-0005] Barnes, S. A. , Young, J. W. , Markou, A. , Adham, N. , Gyertyán, I. , & Kiss, B. (2018). The effects of cariprazine and aripiprazole on PCP‐induced deficits on attention assessed in the 5‐choice serial reaction time task. Psychopharmacology, 235(5), 1403–1414. 10.1007/s00213-018-4857-0 29473089 PMC5920008

[bph14923-bib-0006] Bender, K. J. , Ford, C. P. , & Trussell, L. O. (2010). Dopaminergic modulation of axon initial segment calcium channels regulates action potential initiation. Neuron, 68(3), 500–511. 10.1016/j.neuron.2010.09.026 21040850 PMC2987607

[bph14923-bib-0007] Birnbaum, R. , & Weinberger, D. R. (2017). Genetic insights into the neurodevelopmental origins of schizophrenia. Nature Reviews. Neuroscience, 18(12), 727–740. 10.1038/nrn.2017.125 29070826

[bph14923-bib-0008] Bouthenet, M. L. , Souil, E. , Martres, M. P. , Sokoloff, P. , Giros, B. , & Schwartz, J. C. (1991). Localization of dopamine D_3_ receptor mRNA in the rat brain using in situ hybridization histochemistry: Comparison with dopamine D2 receptor mRNA. Brain Research, 564(2), 203–219. 10.1016/0006-8993(91)91456-b 1839781

[bph14923-bib-0009] Cadinu, D. , Grayson, B. , Podda, G. , Harte, M. K. , Doostdar, N. , & Neill, J. C. (2017). NMDA receptor antagonist rodent models for cognition in schizophrenia and identification of novel drug treatments, an update. Neuropharmacology, S0028‐3908(17), 30584–30581. 10.1016/j.neuropharm.2017.11.045 29196183

[bph14923-bib-0010] Çalışkan, G. , Schulz, S. B. , Gruber, D. , Behr, J. , Heinemann, U. , & Gerevich, Z. (2015). Corticosterone and corticotropin‐releasing factor acutely facilitate gamma oscillations in the hippocampus in vitro. The European Journal of Neuroscience, 41(1), 31–44. 10.1111/ejn.12750 25306895

[bph14923-bib-0011] Cardin, J. A. , Carlén, M. , Meletis, K. , Knoblich, U. , Zhang, F. , Deisseroth, K. , … Moore, C. I. (2009). Driving fast‐spiking cells induces gamma rhythm and controls sensory responses. Nature, 459(7247), 663–667. 10.1038/nature08002 19396156 PMC3655711

[bph14923-bib-0012] Cho, R. Y. , Konecky, R. O. , & Carter, C. S. (2006). Impairments in frontal cortical gamma synchrony and cognitive control in schizophrenia. Proceedings of the National Academy of Sciences of the United States of America, 103(52), 19878–19883. 10.1073/pnas.0609440103 17170134 PMC1750867

[bph14923-bib-0013] Clarkson, R. L. , Liptak, A. T. , Gee, S. M. , Sohal, V. S. , & Bender, K. J. (2017). D_3_ receptors regulate excitability in a unique class of prefrontal pyramidal cells. The Journal of Neuroscience: The Official Journal of the Society for Neuroscience, 37(24), 5846–5860. 10.1523/JNEUROSCI.0310-17.2017 28522735 PMC5473204

[bph14923-bib-0014] Curtis, M. J. , Alexander, S. , Cirino, G. , Docherty, J. R. , George, C. H. , Giembycz, M. A. , … Ahluwalia, A. (2018). Experimental design and analysis and their reporting II: Updated and simplified guidance for authors and peer reviewers. British Journal of Pharmacology, 175(7), 987–993. 10.1111/bph.14153 29520785 PMC5843711

[bph14923-bib-0015] Daniel, D. , Nasrallah, H. , Earley, W. , Durgam, S. , Lu, K. , Szatmári, B. , … Patel, M. (2017). Effects of cariprazine on negative symptoms, cognitive impairment, and prosocial functioning in patients with predominant negative symptoms: Post hoc analysis of a phase III, placebo‐, and active‐controlled study. Schizophrenia Bulletin, 43(Suppl 1), S13 10.1093/schbul/sbx021.036

[bph14923-bib-0016] Dickinson, A. , Bruyns‐Haylett, M. , Jones, M. , & Milne, E. (2015). Increased peak gamma frequency in individuals with higher levels of autistic traits. The European Journal of Neuroscience, 41(8), 1095–1101. 10.1111/ejn.12881 25858292

[bph14923-bib-0017] Faul, F. , Erdfelder, E. , Lang, A.‐G. , & Buchner, A. (2007). G*Power 3: A flexible statistical power analysis program for the social, behavioral, and biomedical sciences. Behavior Research Methods, 39(2), 175–191. 10.3758/bf03193146 17695343

[bph14923-bib-0018] Fisahn, A. , Pike, F. G. , Buhl, E. H. , & Paulsen, O. (1998). Cholinergic induction of network oscillations at 40 Hz in the hippocampus in vitro. Nature, 394(6689), 186–189. 10.1038/28179 9671302

[bph14923-bib-0019] Fries, P. (2009). Neuronal gamma‐band synchronization as a fundamental process in cortical computation. Annual Review of Neuroscience, 32, 209–224. 10.1146/annurev.neuro.051508.135603 19400723

[bph14923-bib-0020] Girgis, R. R. , Slifstein, M. , D'Souza, D. , Lee, Y. , Periclou, A. , Ghahramani, P. , … Rakhit, A. (2016). Preferential binding to dopamine D_3_ over D_2_ receptors by cariprazine in patients with schizophrenia using PET with the D_3_/D_2_ receptor ligand [^11^C]‐(+)‐PHNO. Psychopharmacology, 233(19–20), 3503–3512. 10.1007/s00213-016-4382-y 27525990 PMC5035321

[bph14923-bib-0021] Gonzalez‐Burgos, G. , Cho, R. Y. , & Lewis, D. A. (2015). Alterations in cortical network oscillations and parvalbumin neurons in schizophrenia. Biological Psychiatry, 77(12), 1031–1040. 10.1016/j.biopsych.2015.03.010 25863358 PMC4444373

[bph14923-bib-0022] Grace, A. A. (2016). Dysregulation of the dopamine system in the pathophysiology of schizophrenia and depression. Nature Reviews. Neuroscience, 17(8), 524–532. 10.1038/nrn.2016.57 27256556 PMC5166560

[bph14923-bib-0023] Graff‐Guerrero, A. , Mamo, D. , Shammi, C. M. , Mizrahi, R. , Marcon, H. , Barsoum, P. , … Kapur, S. (2009). The effect of antipsychotics on the high‐affinity state of D_2_ and D_3_ receptors: A positron emission tomography study with [^11^C]‐(+)‐PHNO. Archives of General Psychiatry, 66(6), 606–615. 10.1001/archgenpsychiatry.2009.43 19487625

[bph14923-bib-0024] Gyertyán, I. , Kiss, B. , Sághy, K. , Laszy, J. , Szabó, G. , Szabados, T. , … Szombathelyi, Z. (2011). Cariprazine (RGH‐188), a potent D_3_/D_2_ dopamine receptor partial agonist, binds to dopamine D_3_ receptors in vivo and shows antipsychotic‐like and procognitive effects in rodents. Neurochemistry International, 59(6), 925–935. 10.1016/j.neuint.2011.07.002 21767587

[bph14923-bib-0025] Harding, S. D. , Sharman, J. L. , Faccenda, E. , Southan, C. , Pawson, A. J. , Ireland, S. , … NC‐IUPHAR (2018). The IUPHAR/BPS guide to pharmacology in 2018: Updates and expansion to encompass the new guide to immunopharmacology. Nucleic Acids Research, 46(D1), D1091–D1106. 10.1093/nar/gkx1121 29149325 PMC5753190

[bph14923-bib-0026] Herrmann, C. S. , & Demiralp, T. (2005). Human EEG gamma oscillations in neuropsychiatric disorders. Clinical Neurophysiology, 116(12), 2719–2733. 10.1016/j.clinph.2005.07.007 16253555

[bph14923-bib-0027] Herrmann, C. S. , Munk, M. H. , & Engel, A. K. (2004). Cognitive functions of gamma‐band activity: Memory match and utilization. Trends in Cognitive Sciences, 8(8), 347–355. 10.1016/j.tics.2004.06.006 15335461

[bph14923-bib-0028] Hudson, M. R. , Rind, G. , O'Brien, T. J. , & Jones, N. C. (2016). Reversal of evoked gamma oscillation deficits is predictive of antipsychotic activity with a unique profile for clozapine. Translational Psychiatry, 6, e784 10.1038/tp.2016.51 27093066 PMC4872409

[bph14923-bib-0029] Jentsch, J. D. , & Roth, R. H. (1999). The neuropsychopharmacology of phencyclidine: From NMDA receptor hypofunction to the dopamine hypothesis of schizophrenia. Neuropsychopharmacology, 20(3), 201–225. 10.1016/S0893-133X(98)00060-8 10063482

[bph14923-bib-0030] Kapur, S. (2003). Psychosis as a state of aberrant salience: a framework linking biology, phenomenology, and pharmacology in schizophrenia. The American Journal of Psychiatry, 160(1), 13–23. 10.1176/appi.ajp.160.1.13 12505794

[bph14923-bib-0031] Kehrer, C. , Dugladze, T. , Maziashvili, N. , Wójtowicz, A. , Schmitz, D. , Heinemann, U. , & Gloveli, T. (2007). Increased inhibitory input to CA1 pyramidal cells alters hippocampal gamma frequency oscillations in the MK‐801 model of acute psychosis. Neurobiology of Disease, 25(3), 545–552. 10.1016/j.nbd.2006.10.015 17169567

[bph14923-bib-0032] Khan, Z. U. , Gutiérrez, A. , Martín, R. , Peñafiel, A. , Rivera, A. , & De La Calle, A. (1998). Differential regional and cellular distribution of dopamine D_2_‐like receptors: An immunocytochemical study of subtype‐specific antibodies in rat and human brain. The Journal of Comparative Neurology, 402(3), 353–371. 10.1002/(sici)1096-9861(19981221)402:3<353::aid-cne5>3.0.co;2-4 9853904

[bph14923-bib-0033] Kilkenny, C. , Browne, W. J. , Cuthill, I. C. , Emerson, M. , & Altman, D. G. (2010). Improving bioscience research reporting: The ARRIVE guidelines for reporting animal research. PLoS Biology, 8(6), e1000412 10.1371/journal.pbio.1000412 20613859 PMC2893951

[bph14923-bib-0034] Kiss, B. , Horváth, A. , Némethy, Z. , Schmidt, E. , Laszlovszky, I. , Bugovics, G. , … Szombathelyi, Z. (2010). Cariprazine (RGH‐188), a dopamine D_3_ receptor‐preferring, D_3_/D_2_ dopamine receptor antagonist‐partial agonist antipsychotic candidate: in vitro and neurochemical profile. The Journal of Pharmacology and Experimental Therapeutics, 333(1), 328–340. 10.1124/jpet.109.160432 20093397

[bph14923-bib-0035] Kittelberger, K. , Hur, E. E. , Sazegar, S. , Keshavan, V. , & Kocsis, B. (2012). Comparison of the effects of acute and chronic administration of ketamine on hippocampal oscillations: Relevance for the NMDA receptor hypofunction model of schizophrenia. Brain Structure & Function, 217(2), 395–409. 10.1007/s00429-011-0351-8 21979451 PMC3288729

[bph14923-bib-0036] Krause, M. , & Jia, Y. (2005). Serotonergic modulation of carbachol‐induced rhythmic activity in hippocampal slices. Neuropharmacology, 48(3), 381–390. 10.1016/j.neuropharm.2004.10.011 15721170

[bph14923-bib-0037] Lahti, A. C. , Weiler, M. A. , Tamara Michaelidis, B. A. , Parwani, A. , & Tamminga, C. A. (2001). Effects of ketamine in normal and schizophrenic volunteers. Neuropsychopharmacology, 25(4), 455–467. 10.1016/S0893-133X(01)00243-3 11557159

[bph14923-bib-0038] Lee, S. H. , Kim, D. W. , Kim, E. Y. , Kim, S. , & Im, C. H. (2010). Dysfunctional gamma‐band activity during face structural processing in schizophrenia patients. Schizophrenia Research, 119(1–3), 191–197. 10.1016/j.schres.2010.02.1058 20303713

[bph14923-bib-0039] Lemercier, C. E. , Holman, C. , & Gerevich, Z. (2017). Aberrant alpha and gamma oscillations ex vivo after single application of the NMDA receptor antagonist MK‐801. Schizophrenia Research, 188, 118–124. 10.1016/j.schres.2017.01.017 28109667

[bph14923-bib-0040] Lemercier, C. E. , Schulz, S. B. , Heidmann, K. E. , Kovács, R. , & Gerevich, Z. (2016). Dopamine D_3_ receptors inhibit hippocampal gamma oscillations by disturbing CA3 pyramidal cell firing synchrony. Frontiers in Pharmacology, 6, 297. 26779018 10.3389/fphar.2015.00297 PMC4702013

[bph14923-bib-0041] Lu, C. B. , Jefferys, J. G. , Toescu, E. C. , & Vreugdenhil, M. (2011). In vitro hippocampal gamma oscillation power as an index of in vivo CA3 gamma oscillation strength and spatial reference memory. Neurobiology of Learning and Memory, 95(3), 221–230. 10.1016/j.nlm.2010.11.008 21093596

[bph14923-bib-0042] Manahan‐Vaughan, D. , von Haebler, D. , Winter, C. , Juckel, G. , & Heinemann, U. (2008). A single application of MK801 causes symptoms of acute psychosis, deficits in spatial memory, and impairment of synaptic plasticity in rats. Hippocampus, 18(2), 125–134. 10.1002/hipo.20367 17924525

[bph14923-bib-0043] Mauri, M. C. , Paletta, S. , Di Pace, C. , Reggiori, A. , Cirnigliaro, G. , Valli, I. , & Altamura, A. C. (2018). Clinical pharmacokinetics of atypical antipsychotics: An update. Clinical Pharmacokinetics, 57, 1493–1528. 10.1007/s40262-018-0664-3 29915922

[bph14923-bib-0044] Meltzer, H. Y. , Rajagopal, L. , Huang, M. , Oyamada, Y. , Kwon, S. , & Horiguchi, M. (2013). Translating the *N*‐methyl‐d‐aspartate receptor antagonist model of schizophrenia to treatments for cognitive impairment in schizophrenia. The International Journal of Neuropsychopharmacology/Official Scientific Journal of the Collegium Internationale Neuropsychopharmacologicum (CINP), 16(10), 2181–2194. 10.1017/S1461145713000928 24099265

[bph14923-bib-0045] Mierau, J. , Schneider, F. J. , Ensinger, H. A. , Chio, C. L. , Lajiness, M. E. , & Huff, R. M. (1995). Pramipexole binding and activation of cloned and expressed dopamine D_2_, D_3_ and D_4_ receptors. European Journal of Pharmacology, 290(1), 29–36. 10.1016/0922-4106(95)90013-6 7664822

[bph14923-bib-0046] Miyamoto, S. , Miyake, N. , Jarskog, L. F. , Fleischhacker, W. W. , & Lieberman, J. A. (2012). Pharmacological treatment of schizophrenia: A critical review of the pharmacology and clinical effects of current and future therapeutic agents. Molecular Psychiatry, 17(12), 1206–1227. 10.1038/mp.2012.47 22584864

[bph14923-bib-0047] Mizrahi, R. , Agid, O. , Borlido, C. , Suridjan, I. , Rusjan, P. , Houle, S. , … Kapur, S. (2011). Effects of antipsychotics on D_3_ receptors: A clinical PET study in first episode antipsychotic naive patients with schizophrenia using [^11^C]‐(+)‐PHNO. Schizophrenia Research, 131(1–3), 63–68. 10.1016/j.schres.2011.05.005 21684721

[bph14923-bib-0048] Nakajima, S. , Gerretsen, P. , Takeuchi, H. , Caravaggio, F. , Chow, T. , Le Foll, B. , … Graff‐Guerrero, A. (2013). The potential role of dopamine D₃ receptor neurotransmission in cognition. European Neuropsychopharmacology, 23(8), 799–813. 10.1016/j.euroneuro.2013.05.006 23791072 PMC3748034

[bph14923-bib-0049] Neill, J. C. , Grayson, B. , Kiss, B. , Gyertyán, I. , Ferguson, P. , & Adham, N. (2016). Effects of cariprazine, a novel antipsychotic, on cognitive deficit and negative symptoms in a rodent model of schizophrenia symptomatology. European Neuropsychopharmacology, 26(1), 3–14. 10.1016/j.euroneuro.2015.11.016 26655189

[bph14923-bib-0050] Németh, G. , Laszlovszky, I. , Czobor, P. , Szalai, E. , Szatmári, B. , Harsányi, J. , … Fleischhacker, W. W. (2017). Cariprazine versus risperidone monotherapy for treatment of predominant negative symptoms in patients with schizophrenia: A randomised, double‐blind, controlled trial. Lancet, 389(10074), 1103–1113. 10.1016/S0140-6736(17)30060-0 28185672

[bph14923-bib-0051] Newcomer, J. W. , Farber, N. B. , Jevtovic‐Todorovic, V. , Selke, G. , Melson, A. K. , Hershey, T. , … Olney, J. W. (1999). Ketamine‐induced NMDA receptor hypofunction as a model of memory impairment and psychosis. Neuropsychopharmacology, 20(2), 106–118. 10.1016/S0893-133X(98)00067-0 9885791

[bph14923-bib-0052] Reinhart, R. M. , & Woodman, G. F. (2014). Oscillatory coupling reveals the dynamic reorganization of large‐scale neural networks as cognitive demands change. Journal of Cognitive Neuroscience, 26(1), 175–188. 10.1162/jocn_a_00470 23984947 PMC3990735

[bph14923-bib-0053] Richtand, N. M. , Kelsoe, J. R. , Segal, D. S. , & Kuczenski, R. (1995). Regional quantification of D_1_, D_2_, and D_3_ dopamine receptor mRNA in rat brain using a ribonuclease protection assay. Brain Research. Molecular Brain Research, 33(1), 97–103. 10.1016/0169-328x(95)00112-6 8774950

[bph14923-bib-0054] Rodriguez, E. , George, N. , Lachaux, J. P. , Martinerie, J. , Renault, B. , & Varela, F. J. (1999). Perception's shadow: Long‐distance synchronization of human brain activity. Nature, 397(6718), 430–433. 10.1038/17120 9989408

[bph14923-bib-0055] Schulz, S. B. , Heidmann, K. E. , Mike, A. , Klaft, Z. J. , Heinemann, U. , & Gerevich, Z. (2012). First and second generation antipsychotics influence hippocampal gamma oscillations by interactions with 5‐HT3 and D_3_ receptors. British Journal of Pharmacology, 167(7), 1480–1491. 10.1111/j.1476-5381.2012.02107.x 22817643 PMC3514761

[bph14923-bib-0056] Schulz, S. B. , Klaft, Z. J. , Rösler, A. R. , Heinemann, U. , & Gerevich, Z. (2012). Purinergic P2X, P2Y and adenosine receptors differentially modulate hippocampal gamma oscillations. Neuropharmacology, 62(2), 914–924. 10.1016/j.neuropharm.2011.09.024 22001427

[bph14923-bib-0057] Sokoloff, P. , Giros, B. , Martres, M. P. , Bouthenet, M. L. , & Schwartz, J. C. (1990). Molecular cloning and characterization of a novel dopamine receptor (D_3_) as a target for neuroleptics. Nature, 347(6289), 146–151. 10.1038/347146a0 1975644

[bph14923-bib-0058] Sokoloff, P. , & Le Foll, B. (2017). The dopamine D_3_ receptor, a quarter century later. The European Journal of Neuroscience, 45(1), 2–19. 10.1111/ejn.13390 27600596

[bph14923-bib-0059] Spencer, K. M. , Nestor, P. G. , Perlmutter, R. , Niznikiewicz, M. A. , Klump, M. C. , Frumin, M. , … McCarley, R. W. (2004). Neural synchrony indexes disordered perception and cognition in schizophrenia. Proceedings of the National Academy of Sciences of the United States of America, 101(49), 17288–17293. 10.1073/pnas.0406074101 15546988 PMC535363

[bph14923-bib-0060] Swant, J. , Stramiello, M. , & Wagner, J. J. (2008). Postsynaptic dopamine D_3_ receptor modulation of evoked IPSCs via GABA_A_ receptor endocytosis in rat hippocampus. Hippocampus, 18(5), 492–502. 10.1002/hipo.20408 18240318

[bph14923-bib-0061] Uhlhaas, P. J. , & Singer, W. (2010). Abnormal neural oscillations and synchrony in schizophrenia. Nature Reviews. Neuroscience, 11(2), 100–113. 10.1038/nrn2774 20087360

[bph14923-bib-0062] Uhlhaas, P. J. , & Singer, W. (2015). Oscillations and neuronal dynamics in schizophrenia: The search for basic symptoms and translational opportunities. Biological Psychiatry, 77(12), 1001–1009. 10.1016/j.biopsych.2014.11.019 25676489

[bph14923-bib-0063] Van Tol, H. H. , Bunzow, J. R. , Guan, H. C. , Sunahara, R. K. , Seeman, P. , Niznik, H. B. , & Civelli, O. (1991). Cloning of the gene for a human dopamine D_4_ receptor with high affinity for the antipsychotic clozapine. Nature, 350(6319), 610–614. 10.1038/350610a0 1840645

[bph14923-bib-0064] Watson, D. J. G. , King, M. V. , Gyertyán, I. , Kiss, B. , Adham, N. , & Fone, K. C. F. (2016). The dopamine D₃‐preferring D₂/D₃ dopamine receptor partial agonist, cariprazine, reverses behavioural changes in a rat neurodevelopmental model for schizophrenia. European Neuropsychopharmacology, 26(2), 208–224. 10.1016/j.euroneuro.2015.12.020 26723167

[bph14923-bib-0065] Wiescholleck, V. , & Manahan‐Vaughan, D. (2013). Long‐lasting changes in hippocampal synaptic plasticity and cognition in an animal model of NMDA receptor dysfunction in psychosis. Neuropharmacology, 74, 48–58. 10.1016/j.neuropharm.2013.01.001 23376021

[bph14923-bib-0066] Zarnadze, S. , Bäuerle, P. , Santos‐Torres, J. , Böhm, C. , Schmitz, D. , Geiger, J. R. , … Gloveli, T. (2016). Cell‐specific synaptic plasticity induced by network oscillations. eLife, 5, e14912 10.7554/eLife.14912 27218453 PMC4929000

[bph14923-bib-0067] Zimnisky, R. , Chang, G. , Gyertyán, I. , Kiss, B. , Adham, N. , & Schmauss, C. (2013). Cariprazine, a dopamine D_3_‐receptor‐preferring partial agonist, blocks phencyclidine‐induced impairments of working memory, attention set‐shifting, and recognition memory in the mouse. Psychopharmacology, 226(1), 91–100. 10.1007/s00213-012-2896-5 23079899 PMC3572273

